# Effectiveness of tenofovir and entecavir in nucleos(t)ide analogue-naive chronic hepatitis B: A protocol for systematic review and meta-analysis

**DOI:** 10.1097/MD.0000000000016943

**Published:** 2019-08-23

**Authors:** Mao-bing Chen, Hua Wang, Qi-han Zheng, Xu-wen Zheng, Jin-nuo Fan, Yun-long Ding, Mao-xing Yue

**Affiliations:** a Department of Emergency; b Department of ICU, Wujin People Hospital, The Affiliated Hospital of Jiangsu University, Changzhou; c Department of Neurology, Jingjiang People Hospital, The Seventh Affiliated Hospital of Yangzhou University, Jingjiang, Jiangsu; d The People Liberation Army 306 Hospital, Beijing, P.R. China.

**Keywords:** chronic hepatitis B, entecavir, meta-analysis, nucleos(t)ide analogue-naïve, tenofovir

## Abstract

**Background:**

Chronic hepatitis b (CHB) is a serious problem worldwide. Tenofovir disoproxil fumarate (TDF) and entecavir (ETV) both are first-line drugs for CHB, but there is debate about which is more appropriate in nucleos(t)ide analogue-naive CHB.

**Objective:**

To systematically evaluate the effectiveness and safety of tenofovir and ETV in nucleos(t)ide analogue-naive CHB.

**Methods:**

The Web of Science, PubMed, The Cochrane Library, EMBASE, Clinical Trials, and China National Knowledge Infrastructure databases will be electronically searched to collect randomized controlled trials regarding the comparison between tenofovir and ETV in nucleos(t)ide analogue-naive CHB since the date of database inception to July 2019. Two researchers independently screened and evaluated the obtained studies and extracted the outcome indexes. RevMan 5.3 software will be used for the meta-analysis.

**Result:**

We will provide practical and targeted results assessing the effectiveness and safety of TDF and ETV for nucleos(t)ide analogue-naive CHB patients, try to compare the advantages of TDF and ETV.

**Conclusion:**

The stronger evidence about the effectiveness and safety of TDF and ETV for nucleos(t)ide analogue-naive CHB patients will be provided for clinicians.

**Protocol registration number:**

PROSPERO CRD42019134194.

## 1 Introduction

Chronic hepatitis B (CHB) is indicated when there is continued positivity for the hepatitis B virus (HBV) and the course of the disease exceeds half a year or the date of infection is not known, with clinical manifestations of the disease.^[1]^ The clinical manifestations are asthenia, fear of food, nausea, abdominal distension, liver pain, and other symptoms.^[2]^ The liver is large, moderately hard, and tender. Severe cases can be accompanied by symptoms of chronic liver disease, spider nevus, liver palm, and abnormal liver function.^[3]^ According to the World Health Organization report, >2 billion people have been infected with HBV worldwide, and approximately 240 million of them are chronically infected.^[4]^ The current CHB guidelines recommend tenofovir disoproxil fumarate (TDF) or entecavir (ETV) for the treatment of CHB. As first-line drugs for CHB treatment, they have the common advantages of high antiviral efficacy, good tolerance, and excellent genetic barrier, and it is not easy to develop drug resistance to them.^[5]^

Patients with CHB need long-term antiviral treatment. Currently, there is no clear drug withdrawal guideline for antiviral treatment.^[6]^ It is generally believed that antiviral drugs require long-term or even lifelong oral administration to achieve the goal of controlling CHB.^[7,8]^ Patients often have questions about whether TDF or ETV is more appropriate at the time of initial treatment or in the early stages of CHB and whether TDF is better than ETV in terms of efficacy and safety.^[9,10]^ In this study, the efficacy and safety of TDF and ETV in CHB patients were compared to provide a basis for patients to choose the more appropriate antiviral drug.

Before this study, there were similar systematic analysis articles, but at that time, there were few reliable randomized controlled trials (RCTs). In the past 2 years, relevant RCT literatures have been published in journals. This study collected and analyzed those studies.

## 2 Methods

### 2.1 Design and registration

A meta-analysis will be conducted to evaluate the effectiveness of TDF and ETV in nucleos(t)ide analogue-naive CHB. This protocol has been registered on the International Prospective Register of Systematic Reviews, registration number: CRD42019134194 (https://www.crd.york.ac.uk/PROSPERO). No ethical approval is required since this study used data that will be already in the public domain.

### 2.2 Study selection

#### 2.2.1 Study type

The type of this study will be randomized controlled trials (RCTs).

#### 2.2.2 Study object

Patients with definite CHB and no prior experience with nucleos(t)ide analogue therapy will be included. The following patients will be excluded: patients who are infected with HIV or other hepatotropic viruses; those who have drug-induced liver diseases, alcoholic liver disease or autoimmune liver diseases, tumors, serious complications in the heart, kidney, brain, and other organs; and patients who are pregnant or lactating.

#### 2.2.3 Intervening measure

TDF group: the enrolled patients will be given the conventional dose of tenofovir 300 mg/day orally; ETV group: the enrolled patients will be given the conventional dose of ETV 0.5 g/day orally.

#### 2.2.4 Outcome indicator

The following outcomes will assessed and compared between the TDF and ETV groups: differences in the probability of normalized alanine aminotransferase (ALT) indicators, differences in the probability of HBV-DNA–negative results (undetectable), differences in the probability of hepatitis E antigen clearance (HBeAg clearance), differences in the probability of HBeAg seroconversion, and differences in the probability of adverse effects.

#### 2.2.5 Exclusion criteria

Literature whose data cannot be extracted or utilized; literature on animal experiments; literature reviews, etc.

### 2.3 Data sources and searches

**Figure 1:**
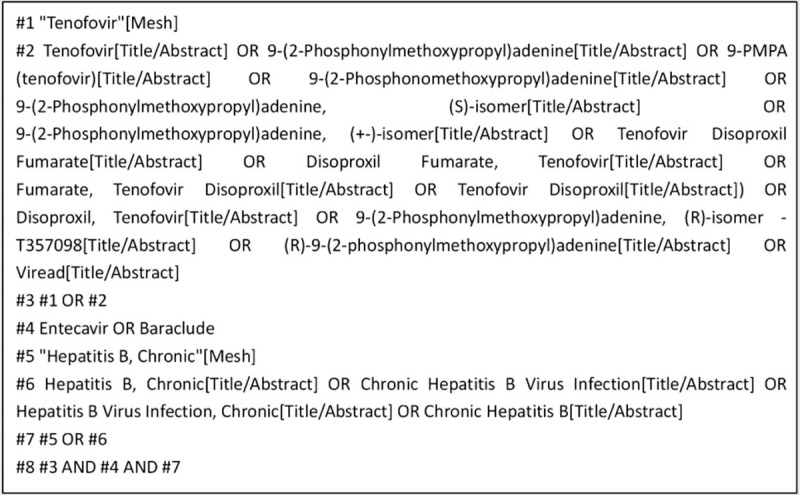
PubMed database retrieval strategy.

We will search English and Chinese language publications through July 2019 using the following databases: Web of Science, PubMed, the Cochrane Library, EMBASE, Clinical Trials, and the China National Knowledge Infrastructure. The search terms included “Tenofovir,” “Entecavir,” and “Hepatitis B, Chronic.” In Figure 1, we use the PubMed database as an example.

### 2.4 Study screening, data extraction, and risk assessment of bias

Data will be collected independently by 2 researchers. The unqualified studies will be eliminated, and the qualified ones will be selected after reading the title, abstract, and full text. Then, the research data will be extracted and checked, and disagreements will be discussed or a decision will be made by the authors. The extracted data include the following: basic information of the study, including title, author, and year of publication; characteristics of the included study, consisting of the study duration, the sample size of the test group and the control group, and the intervention measures; the outcome indicators and data; and the information needed to assess the risk of bias. The risk of bias in the included studies will be assessed using the RCT bias risk assessment tool recommended in the Cochrane Handbook for Systematic Reviews of Interventions (5.1.0).

### 2.5 Statistical analysis

RevMan 5.3 software will be used for the meta-analysis. The dichotomous variables will be expressed as the relative risk as an effect indicator, the continuous variables will be expressed as the mean difference as the effect indicator, and the estimated value and 95% confidence interval will be included as effect analysis statistics. A heterogeneity test will be conducted with the results of each study. The fixed effect model will be used for the analysis if there was no statistical heterogeneity among the results (*I*^2^ ≤ 50%). The sources of heterogeneity need to be analyzed if there is statistical heterogeneity among the results (*I*^2^ > 50%). After excluding the influence of obvious clinical heterogeneity, the random effects model will be used for the analysis. The significance level sets at α = 0.05.

### 2.6 Subgroup analysis

Subgroups will be established based on differences in duration of medication.

### 2.7 Assessment of publication bias

If >10 articles are available for quantitative analysis, we will generate funnel plots to assess publication bias. A symmetrical distribution of funnel plot data indicates that there is no publication bias; otherwise, we will analyze the possible cause and give reasonable interpretation for asymmetric funnel plots.

### 2.8 Confidence in cumulative evidence

GRADE system will be used for assessing the quality of our evidence. According to the grading system, the level of evidence will be rated high, moderate, low, and very low.^[11]^

## 3 Discussion

Tenofovir and ETV are first-line drugs for CHB, and their efficacy and safety are widely recognized.^[12]^ It is, however, difficult to choose between tenofovir or ETV for patients who are initially diagnosed with CHB.

Our goal in treating CHB is to achieve a clinical cure or clinical control. The best result is hepatitis B surface antibody turn positive, and very few people can achieve this.^[13]^ HBV-DNA conversion and ALT normalization are always identified as clinical cure, and HBeAg clearance or HBeAg seroconversion can be considered indications that CHB is under control.^[14]^ So in this study we choose normalized ALT indicator, HBV-DNA negative results (undetectable), HBeAg clearance, and HBeAg seroconversion to assess the effectiveness of TDF and ETV.

Tenofovir is a nucleotide reverse transcriptase inhibitor that inhibits reverse transcriptase in a similar way to nucleoside reverse transcriptase inhibitors and thus has potential anti-HBV activity.^[15]^ Tenofovir bisphosphonates, the active component of tenofovir, inhibit the viral polymerase by directly competing with the natural deoxyribose substrates and terminating DNA strands by inserting DNA.^[8,16]^ ETV is a guanine nucleoside analogue, and its antiviral pharmacological action is similar to that of tenofovir.^[17,18]^

This study will conduct a meta-analysis of related RCTs, provide evidence on the effectiveness of TDF and ETV in CHB treatment, and compare the advantages and disadvantages of TDF and ETV, so as to better guide clinical practice.

## Author contributions

**Conceptualization:** Mao-bing Chen, Qi-han Zheng, Xu-wen Zheng, Yun-long Ding.

**Data curation:** Mao-bing Chen, Hua Wang.

**Formal analysis:** Hua Wang, Mao-xing Yue.

**Funding acquisition:** Mao-xing Yue.

**Investigation:** Hua Wang.

**Methodology:** Mao-bing Chen, Yun-long Ding.

**Project administration:** Jin-nuo Fan.

**Resources:** Xu-wen Zheng.

**Software:** Mao-bing Chen, Yun-long Ding.

**Writing – original draft:** Mao-bing Chen, Hua Wang, Qi-han Zheng, Xu-wen Zheng, Jin-nuo Fan.

**Writing – review and editing:** Mao-bing Chen.

Mao-bing Chen orcid: 0000-0001-5037-9870.
